# Supplementary figures and images for: Integrative growth physiology and transcriptome profiling of probiotic Limosilactobacillus reuteri KUB-AC5
Source: PeerJ. 2021 Oct 5;9:e12226. doi: 10.7717/peerj.12226 (PMC8500091; doi:10.7717/peerj.12226)

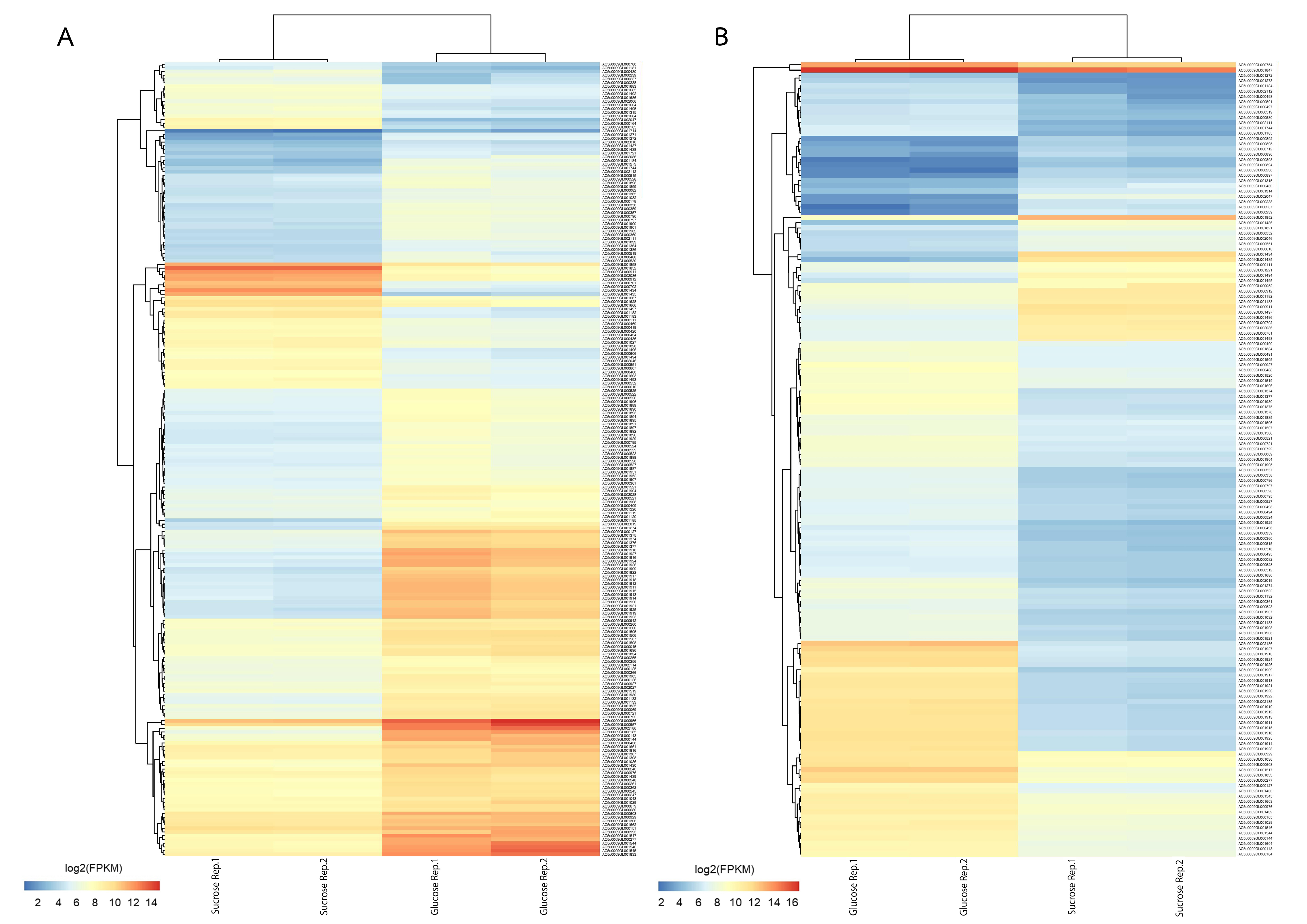

Supplement: Supplemental Information 2 — (A) at L-phase of growth and (B) S-phase of growth. Each gene is colored by log2 FPKM value. [file peerj-09-12226-s002.jpg]
